# Methylmercury Alters the Activities of Hsp90 Client Proteins, Prostaglandin E Synthase/p23 (PGES/23) and nNOS

**DOI:** 10.1371/journal.pone.0098161

**Published:** 2014-05-22

**Authors:** Samuel Caito, Heng Zeng, Judy L. Aschner, Michael Aschner

**Affiliations:** 1 Department of Molecular Pharmacology, Albert Einstein College of Medicine, Bronx, New York, United States of America; 2 Department of Pediatrics, Vanderbilt University School of Medicine, Nashville, Tennessee, United States of America; 3 Department of Pediatrics and Obstetrics & Gynecology and Women's Health, Albert Einstein College of Medicine of Yeshiva University and Children's Hospital at Montefiore, Bronx, New York, United States of America; 4 The Kennedy Center, Albert Einstein College of Medicine, Bronx, New York, United States of America; University of Pecs Medical School, Hungary

## Abstract

Methylmercury (MeHg) is a persistent pollutant with known neurotoxic effects. We have previously shown that astrocytes accumulate MeHg and play a prominent role in mediating MeHg toxicity in the central nervous system (CNS) by altering glutamate signaling, generating oxidative stress, depleting glutathione (GSH) and initiating lipid peroxidation. Interestingly, all of these pathways can be regulated by the constitutively expressed, 90-kDa heat shock protein, Hsp90. As Hsp90 function is regulated by oxidative stress, we hypothesized that MeHg disrupts Hsp90-client protein functions. Astrocytes were treated with MeHg and expression of Hsp90, as well as the abundance of complexes of Hsp90-neuronal nitric oxide synthase (nNOS) and Hsp90-prostaglandin E synthase/p23 (PGES/p23) were assessed. MeHg exposure decreased Hsp90 protein expression following 12 h of treatment while shorter exposures had no effect on Hsp90 protein expression. Interestingly, following 1 or 6 h of MeHg exposure, Hsp90 binding to PGES/p23 or nNOS was significantly increased, resulting in increased prostaglandin E_2_ (PGE_2_) synthesis from MeHg-treated astrocytes. These effects were attenuated by the Hsp90 antagonist, geldanmycin. NOS activity was increased following MeHg treatment while cGMP formation was decreased. This was accompanied by an increase in •O_2_^−^ and H_2_O_2_ levels, suggesting that MeHg uncouples NO formation from NO-dependent signaling and increases oxidative stress. Altogether, our data demonstrates that Hsp90 interactions with client proteins are increased following MeHg exposure, but over time Hsp90 levels decline, contributing to oxidative stress and MeHg-dependent excitotoxicity.

## Introduction

Methylmercury (MeHg) is a potent neurotoxicant. Studies from mass poisonings in Japan and Iraq, as well as the examination of seafood-rich diets of the Seychelles and Faroe Islands, have illustrated the effects of MeHg on human populations [1], [2]. MeHg causes focal lesions, such as loss of cerebellar granular cells and occipital lobe damage, and during extreme poisonings can lead to ataxia, numbness of extremities, muscle weakness, vision and hearing problems, and paralysis in adults [3]. Developmental exposure to MeHg leads to microcephaly and inhibition of neuronal migration, distortion of cortical layers, cerebellar abnormalities, alterations in neurotransmitter systems and alterations in glial cells [3]. Astrocytes, in particular, are highly susceptible to the toxic effects of MeHg. Astrocytes are a preferred cellular site for MeHg accumulation and it has been shown that MeHg-induced neuronal dysfunction is secondary to astrocytic disturbances [4], [5], [6], [7]. MeHg inhibits astrocytic uptake systems for cysteine and cystine, glutamate and aspartate, compromising the cell's redox potential, attenuating glutathione (GSH) content, increasing extracellular glutamate concentrations and sensitizing neurons for excitotoxic injury [8], [9], [10], [11], [12].

MeHg is a potent inducer of oxidative stress through the generation of reactive oxygen species (ROS). H_2_O_2_, superoxide anion (**_ ˙_**O_2_–) and nitric oxide (NO) levels are increased in cultured neurons, astrocytes and in rodent brains following MeHg exposure [13], [14]. Putative mechanisms of MeHg-induced free radical generation include inhibition of mitochondrial electron transport chain complexes II and III, alterations in antioxidant enzyme function, and activation of nitric oxide synthase (NOS) [15], [16], [17]. Additionally, as an electrophile, MeHg reacts readily with thiol and selenol groups on proteins as well with GSH, leading to inhibition of enzymes and alteration of protein structure. This has been described for both glutathione peroxidase and thioredoxin antioxidant enzymes [18], [19]. Altering antioxidant activity and depletion of GSH leads to a more oxidized environment and further generation of ROS, creating a vicious cycle. It is unknown how MeHg affects the 90-kDa heat shock protein (Hsp90), an important regulator of cellular redox status.

Hsp90 is one of the most abundant proteins in eukaryotic cells, constituting 1–2% of total intracellular protein [20]. It is constitutively and ubiquitously expressed and is the most abundant molecular chaperone protein. Chaperones help to achieve and maintain the conformational status of cellular proteins and enzyme complexes by influencing higher order protein structure. Hsp90 protects its client proteins from degradation, which is in contrast to other chaperones, such as Hsp70, which tend to direct misfolded proteins to degradation [21]. Hsp90 is involved in the conformational regulation of key proteins in multiple signaling pathways, including kinases, phosphatases, steroid hormone receptors, nitric oxide synthases and prostaglandin E synthase/p23 (PGES/p23) [22], [23], [24], [25], many of which are involved in MeHg-induced neurotoxicity. Hsp90 contains two key reactive cysteine residues, which along with the stabilizing molybdate, regulates the redox status of client proteins by assisting in the formation and breakage of disulfide bridges [26]. Oxidizing conditions impair the chaperone activity and lead to cleavage of Hsp90, which can cause client protein degradation and cell death [26], [27], [28]. Therefore, we hypothesized that MeHg inhibits Hsp90-client protein interactions and consequently disrupts Hsp90 client protein functions. We tested this hypothesis by measuring Hsp90 expression and protein-protein interactions between Hsp90 and its client proteins, PGES/p23 and nNOS in astrocytes following MeHg exposure. Additionally, we examined prostaglandin E_2_ (PGE_2_) and NO levels as measures of PGES/p23 and nNOS enzymatic functions, respectively.

## Materials and Methods

### Reagents

Unless otherwise stated, all biochemical reagents used in this study were purchased from Sigma Chemicals (St. Louis, MO, USA).

### Primary cell culture and treatments

Primary astrocytes were isolated from cortical tissue of postnatal day-1 neonatal Sprague-Dawley rats, as previously described [29], [30]. After dissociation, centrifugation and resuspension, the mixed glial cell culture was maintained in 225 cm^2^ tissue culture treated flasks fed with minimum essential medium (MEM; Invitrogen, Carlsbad, CA), supplemented with 10% heat-inactivated horse serum (Invitrogen), 100 U/ml penicillin and 100 µg/ml streptomycin. The cultures were maintained in a humidified incubator at 37°C of 5% CO_2_. The media were changed twice a week. After 2 weeks in culture, when cells reached ∼95% confluence, microglia and astrocytes were separated by gentle shaking for 20 min at room temperature. Using this protocol, our lab has achieved ≥95% purity of the isolated astrocytes, as verified by immunostaining for glial fibrillary acidic protein (GFAP) [29], [30]. Equal densities of astrocytes were plated in poly-L-lysine coated plates (BD Biocoat, San Jose, CA). Cells were treated with MeHgCl (ICN Biomedicals, Costa Mesa, CA) in a concentration-dependent (1, 5, or 10 µM) or time-dependent (10 min to 24 h) manner using concentrations that have been shown not to significantly alter cytotoxicity [30], [31]. To examine the effects of COX-1/2 inhibition, Hsp90 antagonism, and glutathione (GSH) depletion, astrocytes were pretreated with indomethacin (0.01 mM x 15 min), geldanamycin. (1 µM x 30 min), and a GSH synthesis inhibitor, buthionine sulfoximine (BSO; 100 mM x 24 h), respectively, before MeHg treatment. To stimulate nNOS expression from astrocytes, cells were treated with LPS (1 µg/ml x 18–24 h) and IFNγ (100 u/ml x 18–24 h) prior to MeHg treatment.

### Western Blot Analysis

Following MeHg treatments, cells were scraped and collected in radioimmunoprecipitation assay (RIPA) buffer with protease inhibitor cocktail on ice. Protein concentrations of the cell lysates were determined using the bicinchoninic acid (BCA) assay (Pierce, Rockford, IL). Twenty µg of protein were loaded onto a 10% SDS-PAGE acrylamide gel. Proteins were electroblotted onto polyvinylidene difluoride membranes, blocked with 0.1% Tween PBS with 5% nonfat milk, and western blots were performed with the primary antibodies anti-Hsp90a/b (sc-13119, Santa Cruz Biotechnology, Santa Cruz, CA), anti-PGES/p23 (Cayman Chemical Co, Ann Arbor, MI, or GeneTex, Inc., San Antonio. TX), anti-nNOS, and anti-iNOS (BD Biosciences, San Jose, CA or MyBioSource, Inc. San Diego, CA), and β-actin (A5316, Sigma). Proteins were visualized by species-appropriate secondary antibodies labeled with horseradish peroxidase (Santa Cruz Biotechnology) and chemiluminescent substrate (ECL; Amersham Pharmacia Biotech).

### Immunofluorescence

Primary astrocytes were grown on 8-well chamber slides (1×104 cells/well), treated with MeHg (1 or 5 µM), and then fixed in 4% paraformaldehyde for 10 min. The cells were then permeabilized for 10 min in 0.3% Triton X-100 in PBS, and blocked for 1 h using 10% normal goat serum in PBS. Samples were incubated with antibodies specific for Hsp90 in a humidified chamber overnight. The primary antibody was detected with an anti-mouse secondary antibody. Nuclei were stained with SYTOX Green (Life Technologies, Grand Island, NY). The coverslips were mounted onto the slides using VectaShield (Vector Laboratories, Burlingame, CA, USA) and viewed under a Nikon Ellipse 80i microscope (Nikon, Tokyo, Japan).

### Co-immunoprecipitation Assay

To examine protein-protein interactions with Hsp90, 500 µg of protein were precleared with pansorbin and then incubated for 6 h at 4°C with either anti-nNOS or anti-Hsp90 antibodies (1 µg/mg total cell protein). Immune complexes were precipitated by overnight incubation at 4°C with protein G-sepharose. The next morning, beads were then washed in lysis buffer and pelleted to remove all unbound protein. The immunoprecipitated samples were heated at 80°C for 15 min in Laemmli loading buffer, and proteins were resolved by SDS-PAGE as described above.

### PGE2 and cGMP Quantification

Levels of PGE_2_ release into cell culture media and cGMP from protein extracts of astrocytes treated with MeHg were measured using PGE_2_ and cGMP enzyme immune assays (EIA; Cayman Chemical, Ann Arbor, MI), respectively, according to the manufacturer's instructions. The developed chromogen absorbance was measured at 405 nm using a VMAX 96-well plate spectrophotometer (Molecular Devices).

### Estimation of RONS Formation

To quantify the effect of MeHg on astrocyte reactive oxygen and nitrogen species, three fluorescent dyes were applied to the cells. For the detection of NO, 20 µM 4,5-diaminofluorescein diacetate (DAF2DA; Molecular Probes, Grand Island, NY) was applied to cells following MeHg treatment (30 min with 10 µM MeHg). In the presence of •NO and oxygen, the non-fluorescent DAF2 is converted to the green fluorescent triazole, DAF2T. After addition of DAF2DA, fluorescence images were taken at an excitation wave length of 488 nm and emission wave length of 515 nm. To assess •O_2_^−^ production, cells were loaded with hydroethidium (10 µM), and treated with 10 mM MeHg for 20 min. Images from the hydroethidium and DAF2DA staining were obtained by confocal microscopy (LSM510; Zeiss 63X water immersion objective, Carl Zeiss MicroImaging, Inc). The resultant confocal data were quantitatively analyzed using the Zeiss LSM software (Carl Zeiss MicroImaging, Inc). To assess H_2_O_2_ production astrocytes were loaded for 30 min with 50 µM 2′,7′-dichlorodihydrofluorescein diacetate (H_2_DCFDA, Life Technologies) before treatment with MeHg (5, 10, 20 µM) in HEPES buffer, and the fluorescence intensity was monitored using a multiwell fluorescence plate reader, as previously described [32].

### Statistical Analysis

All results were expressed as means ± standard errors with a minimum of three independent experiments. One-way analysis of variance (ANOVA) and two-way ANOVA followed by Bonferroni's *post hoc* test were performed using Prism 4 (Graphpad Software Inc., San Diego, CA). Values of P<0.05 were considered statistically significant.

## Results

### MeHg alters Hsp90 protein expression

**Figure 1:**
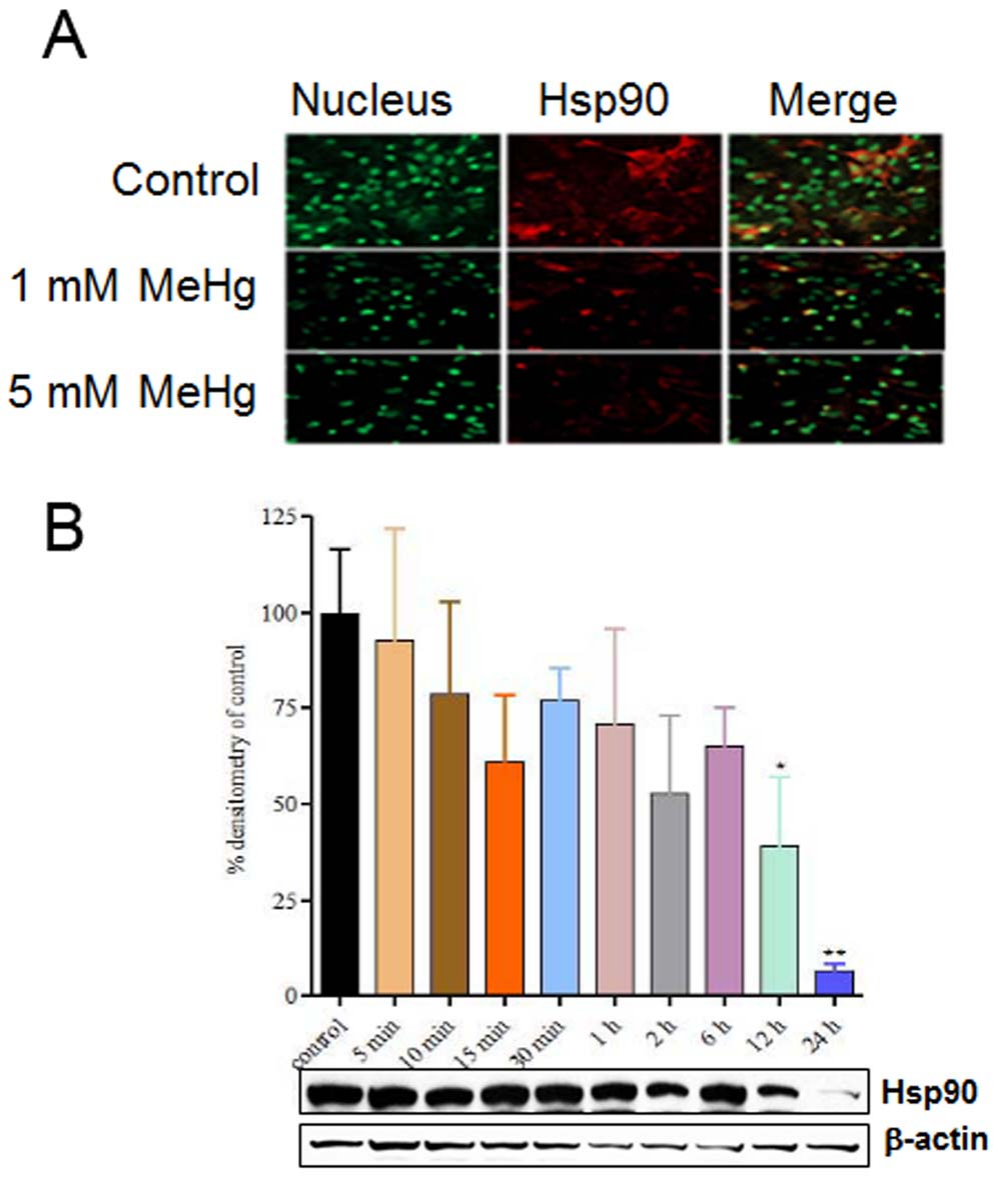
MeHg alters Hsp90 protein expression. (A) Neonatal rat primary astrocytes were treated for 1 h with 1 or 5 µM MeHg. Cells were fixed with 4% paraformaldehyde and used for immunostaining with an anti-Hsp90 monoclonal antibody (red) and Sytox (green) nuclear dye. (B) Astrocytes were treated with 1 µM MeHg for 5, 10, 15, 30 min, 1, 2, 6, 12, or 24 h. Whole cell extracts were used for immunoblot analysis for Hsp90. Results are the mean ± SEM from 4-6 separate astrocyte preparations. *p<0.05, **p<0.01 vs. control.

As MeHg is a strong electrophile and Hsp90 is abundant in cysteine residues, we first examined the direct effect of MeHg on Hsp90 protein levels. Neonatal rat primary astrocytes were treated for 1 h with 1 or 5 µM MeHg. Cells were then stained for Hsp90 by immunofluorescence. Nuclei were stained with Sytox dye. Treatment with MeHg caused a concentration-dependent decrease in Hsp90 immunoflorescence (Figure 1A). Similarly, Western blot analysis of whole-cell extracts from astrocytes treated with 1 µM MeHg showed significant loss of Hsp90 protein levels following 12 and 24 h of exposure (Figure 1B). Hsp90 protein expression was not significantly different from control for exposures under 12 h.

### MeHg increases PGE_2_ production by altering the binding of Hsp90 to PGES/p23

**Figure 2:**
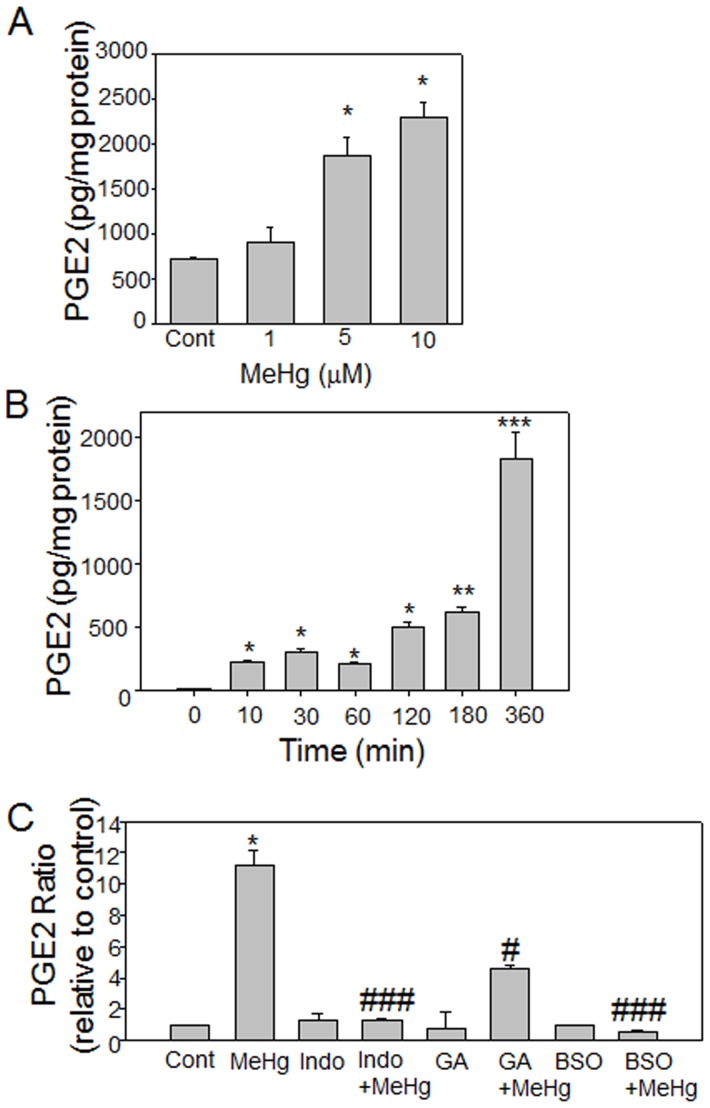
MeHg increases PGE_2_ levels. Astrocytes were treated with (A) 1, 5 and 10 µM MeHg for 3 h or (B) 10 µM MeHg for 10, 20, 60, 120, 180, or 360 min. PGE_2_ levels were measured by enzyme immunoassay. Results are the mean±SEM from 2 different cultures performed in triplicate. *p<0.05, **p<0.01, ***p<0.001 vs. controls. (C) Astrocytes were pretreated with indomethacin (Indo, 0.01 mM) for 15 min, geldanamycin (GA, 1 µM) for 30 min, or buthionine sulfoximine (BSO, 100 µM) for 24 h before treatment with 10 µM MeHg for 3 h. The figure represents a composite of multiple experiments; PGE_2_ in each treatment group is expressed as the ratio to its intra-study control (cont). The effects of COX inhibition and GSH depletion were assessed in 3 different astrocyte cultures performed in triplicate; the effect of Hsp90 antagonism with GA was determined in 2 different cultures performed in triplicates. *p<0.01 vs. control; ^#^ p<0.05, ^###^<0.001 vs. MeHg treatment.

Previously, we reported that MeHg causes time- and concentration-dependent release of arachidonic acid (AA) from astrocytes, associated with increased expression of phospholipase A_2_ (cPLA_2_) mRNA and protein [33]. This effect was fully reversed by treatment with the specific cPLA_2_ inhibitor, AACOCF_3_, suggesting a direct effect of MeHg on cPLA_2_ [33]. Here we examined a downstream effect of AA release, namely the formation of PGE_2_. PGE_2_ levels were first measured in cell culture media from astrocytes treated with 1, 5, or 10 µM MeHg for 3 h. Treatment with 1 µM MeHg had no significant effect, but both 5 and 10 µM MeHg treatments increased PGE_2_ release when compared to untreated cells (Figure 2A). Analogous dose-dependent effects of MeHg were seen following 1 and 6 hrs exposures (data not shown). We next measured PGE_2_ release from astrocytes treated with 10 µM MeHg over the time course of 10 min to 360 min (Figure 2B). We have previously shown that MeHg increases AA release from astrocytes over a similar time course [33]. MeHg significantly increased PGE_2_ levels at all time points examined, with the highest release at 360 min (Figure 2B).

PGE_2_ is formed by the actions of cyclooxygenase-1 (COX-1) and PGE_2_ synthase (PGES/p23). PGES/p23 requires glutathione (GSH) for optimal activity and is a Hsp90 cofactor [34]. PGE_2_ release was measured from astrocytes treated with 10 µM MeHg for 3 hours in the presence of COX-1/2 inhibitor indomethacin (Indo, 0.01 mM), Hsp90 antagonist geldanamycin (GA, 1 µM), or GSH depletion agent, buthionine sulfoximine (BSO, 100 mM) (Figure 2C).

Inhibition of COX-1/2 by indomethacin abolished MeHg-induced PGE_2_ release (Figure 2C). Likewise, BSO inhibited MeHg-stimulated PGE_2_ production. GA significantly decreased the amount of PGE_2_ released from the astrocytes upon MeHg treatment (Figure 2C), suggesting that Hsp90 function is important for MeHg-induced PGE_2_ synthesis and release.

**Figure 3:**
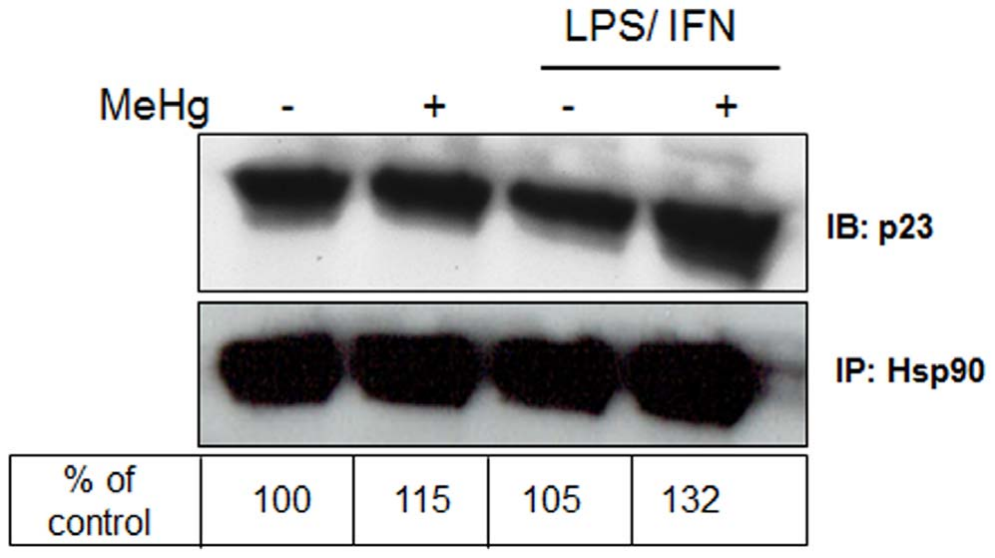
Interaction of Hsp90 and PGES/p23 is promoted by MeHg. Hsp90 was immunoprecipitated from whole cell extracts of astrocytes treated for 1 µM MeHg, 18 h with LPS (1 µµg/ml)/IFNγ (100 u/ml), or both 18 h LPS/INFγ and 1 h MeHg. Equal amounts of immunoprecipitated Hsp90 was used for immunoblotting of PGES/p23. Blot is representative of three separate experiments. Percent change in the amount of p23 relative to Hsp90 is shown below the blot, where basal levels of the interaction are designated 100% and increases in the interaction are values above 100.

As PGES/p23 is known to interact with Hsp90 we examined whether MeHg alters this interaction to promote PGE_2_ synthesis. Astrocytes were treated with 10 µM MeHg for 1 or 6 h in the presence or absence of an 18–24 h pre-treatment with LPS (1 µg/ml)/IFNγ (100 u/ml). LPS/INFγ activates astrocytes to upregulate glial fibrillary acidic protein (GFAP), iNOS and nNOS, as well as release proinflammatory, neurotrophic and neurotoxic mediators [35], [36]. After MeHg treatment, Hsp90 was immunoprecipitated from whole-cell protein extracts and levels of PGES/p23 were examined by immunoblot. Following 1 h MeHg treatment there was increased binding of Hsp90 and PGES/p23 when astrocytes were activated with LPS/IFNγ (Figure 3). The reverse experiment (immunoprecipitation with anti-PGES/p23 antibody and immunoblotting with anti-Hsp90 antibody) also confirmed markedly enhanced Hsp90/PGES binding following 6 h MeHg exposure (data not shown).

### MeHg alters the association between Hsp90 and neuronal nNOS and iNOS

**Figure 4:**
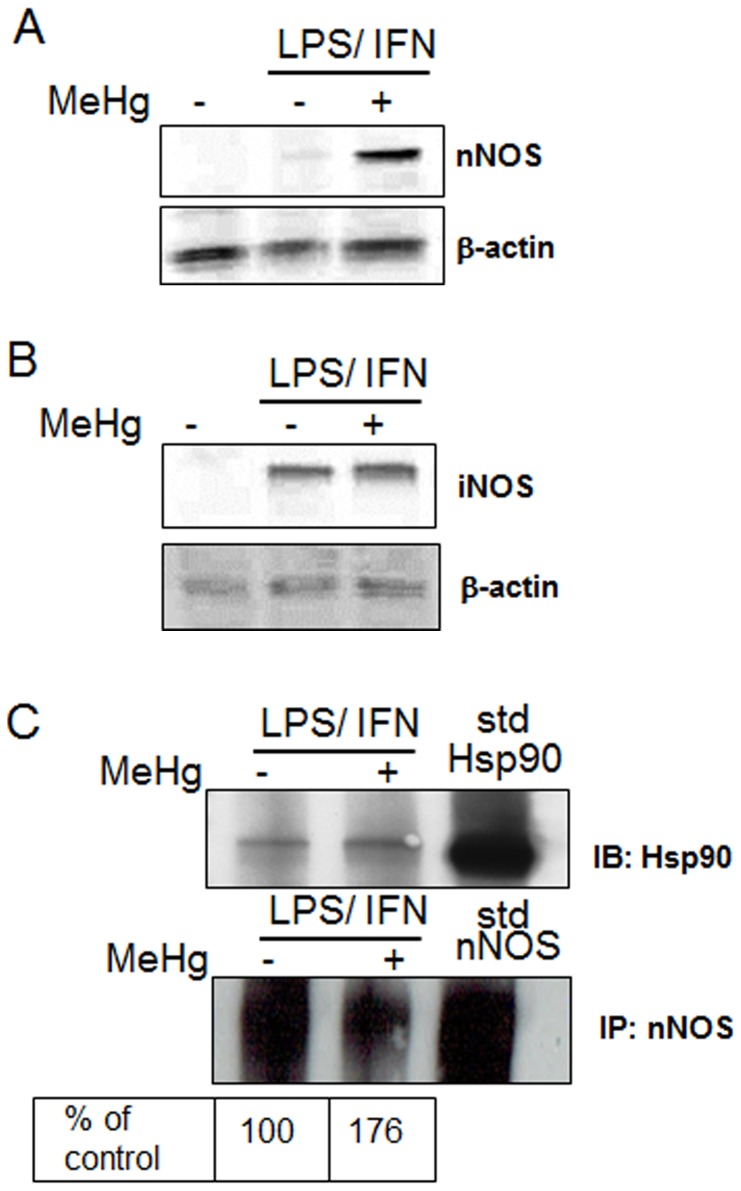
MeHg increases nNOS and iNOS levels. Astrocytes were treated with LPS (1 µg/ml)/IFNγ (100 u/ml) for 18 h and/or 10 µM MeHg for 6 h. Levels of (A) nNOS and (B) iNOS were measured by western blot analysis. (C) nNOS was immunoprecipitated from whole cell extracts of astrocytes stimulated with LPS (1 µg/ml)/IFNγ (100 u/ml) for 18 h alone or followed by 10 µM MeHg for 6 h. Equal amount of immunoprecipitated nNOS was used for immunoblotting of Hsp90. Protein standards for both Hsp90 and nNOS were loaded into the same lane of the gel. Blot is representative of three separate experiments. Percent change in the amount of Hsp90 relative to nNOS is shown below the blot, where basla levels of the interaction are designated 100% and increases in the interaction are values above 100.

Given the observation that MeHg increases the association between Hsp90 and PGES/p23, we were interested in whether MeHg influences other Hsp90-client protein interactions, particularly with nNOS. Astrocytes were activated with LPS (1 µg/ml) and IFNγ (100 u/ml) for 18–24 h before treatment with 10 µM MeHg for 1 h. LPS/IFNγ increased both nNOS and iNOS expression (Figure 4 A and B). MeHg treatment increased nNOS expression after LPS/IFNγ stimulation (Figure 4A), but did not change iNOS expression (Figure 4B), suggesting that nNOS is more responsive to MeHg. We next examined whether MeHg affected Hsp90 binding to nNOS. nNOS was immunoprecipitated from protein lysates of astrocytes treated with LPS (1 µg/ml) and IFNγ (100 u/ml) for 18 h before treatment with 10 µM MeHg for 6 h, and Hsp90 was immunoblotted. Hsp90 and nNOS co-immunoprecipitate in the presence of LPS/IFNg, and this binding complex was increased following MeHg exposure (Figure 4C).

### MeHg uncouples NOS activity and increases NOS-derived superoxide (•O_2_^−^) production

**Figure 5:**
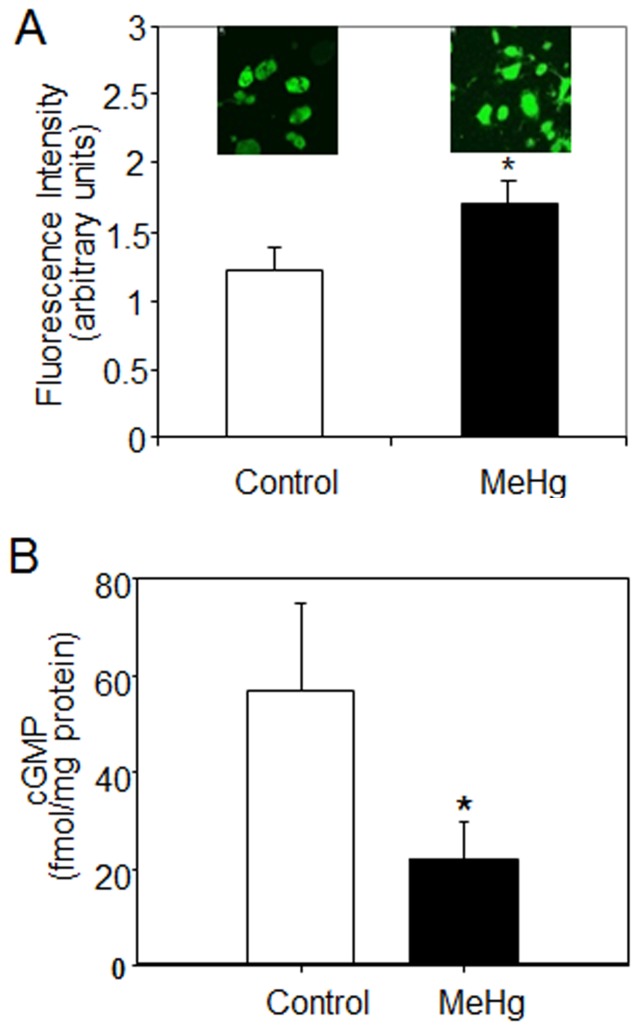
Disruption of NO signaling by MeHg. (A) Astrocytes were treated for 30 min with 10 µM MeHg. After addition of 20 µM DAF2DA, fluorescence images were taken and fluorescence intensity was calculated. Results are mean ± SEM from 3 independent experiments. *p<0.05 vs. control. (B) cGMP concentrations were measured by enzyme immunoassay from astrocytes treated for 1 h with 10 µM MeHg. Results are mean ± SEM from 4–6 separate astrocyte preparations. *p<0.05 vs. control.

Since association of Hsp90 with nNOS enhances nNOS activity [37], we next determined whether there was increased NO signaling immediately following MeHg treatment. Release of NO was assessed in astrocytes treated with 10 µM MeHg for 30 min and then loaded with 20 µM 4,5-diaminofluorescein diacetate (DAF2DA). Fluorescent images were taken 20 min following addition of the DAF2DA. Astrocytes treated with MeHg showed significantly higher levels of NO than control cells (Figure 5A). NO activates guanylate cyclase to produce cGMP as a second messenger in various cell signaling pathways. Therefore, we quantified cGMP levels in astrocytes treated with 10 µM MeHg for 1 h using an enzyme immunoassay. cGMP levels were reduced by ∼50% in astrocytes treated with MeHg relative to controls (Figure 5B). An increase in NO released by the cells but a dampening of the NO-dependent second messenger, cGMP, suggests that MeHg uncouples NO release from downstream NO-dependent signaling.

**Figure 6:**
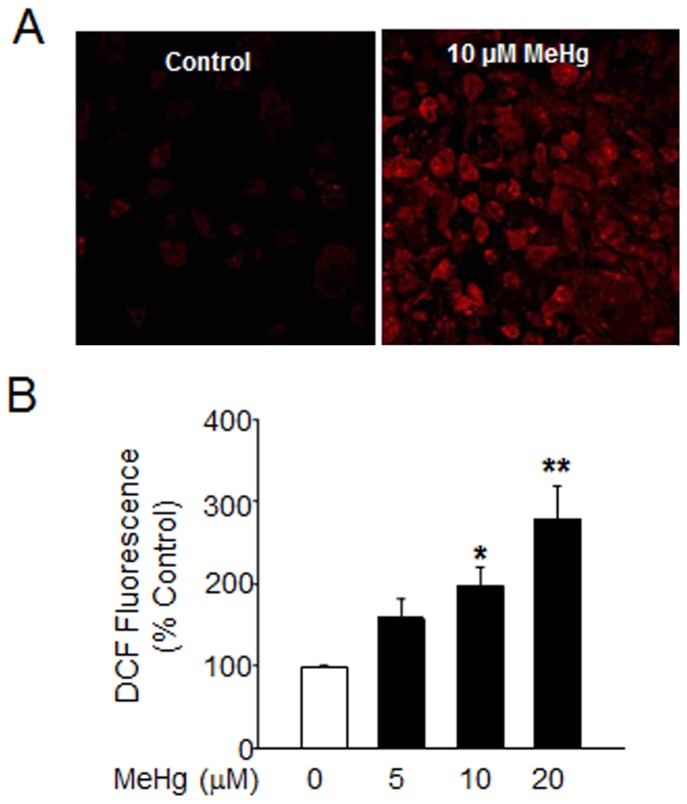
MeHg increases ROS in astrocytes. (A) To assess .O_2_^−^ production, astrocytes were exposed to hydroethidium and then treated with 10 µM MeHg for 20 min. Images are representative of 3 independent experiments. (B) H_2_O_2_ production was assessed by loading. astrocytes with 50 µM 2′,7′-dichlorodihydrofluorescein for 30 min. Astrocytes were then treated with 5, 10, and 20 µM MeHg for 1 h and DCF fluorescence was measured. Results are depicted as % DCF fluorescence in control astrocytes. Results are mean ± SEM from 4 separate astrocyte preparations. *p<0.05 vs. control.

Under conditions of substrate depletion and certain pathological conditions, such as hypertension and atherosclerosis, nNOS has been shown to produce ROS, particularly H_2_O_2_ and •O_2_^−^ [38], [39]. •O_2_^−^ production was assessed by confocal microscopy of hydroethidium-loaded astrocytes exposed to 10 µM MeHg for 20 min. MeHg significantly increased the amount of ethidium fluorescence (Figure 6A). H_2_O_2_ is formed from •O_2_^−^ in the presence of superoxide dismutase and catalase. To examine H_2_O_2_ formation, we preloaded astrocytes with 50 µM 2′,7′-dichlorodihydrofluorescein for 30 min and then treated with 5, 10, and 20 µM MeHg for 1 h. DCF fluorescence was significantly increased in astrocytes treated with 10 and 20 µM MeHg as compared to control (Figure 6B). Together, these data demonstrate that MeHg increases several ROS produced in astrocytes, including the ROS, NO which can interact •O_2_^−^ to form the highly toxic peroxynitrite (OONO^−^) [40].

## Discussion

MeHg is a known neurotoxin that has been shown to affect astrocytes as well as neurons, leading to cellular dysfunction and neuronal death. We have previously shown that MeHg induces astrocytic swelling triggering glutamate release and inhibits the uptake of cystine and cysteine [41], [42], [43], [44], [45], reducing the astrocytes' ability to synthesize GSH and buffer ROS. Increased glutamate release stimulates N-methyl D-aspartate (NMDA) receptors on neurons increasing intracellular Ca^2+^ concentrations, which leads to the activation of nNOS and mitochondrial dysfunction [46]. MeHg also has been shown to activate cPLA2, possibly through increased Ca^2+^ concentrations, releasing AA, and ultimately leading to protein carbonylation and synthesis of prostaglandins and isoprostanes [33], [47]. Central to both prostaglandin synthesis and nNOS activation pathways is regulation by the Hsp90 chaperone. Our data are the first to describe that MeHg alters Hsp90 chaperone function with subsequent activation of nNOS and PGES/p23, leading to increased release of PGE_2_ and ROS from astrocytes.

The neuroprotective roles of heat shock chaperone proteins are well recognized. Both Hsp70 and Hsp90 protect neurons from thermal injury, ischemia, protein aggregation, and apoptosis [48], [49], [50]. Hsp90 can also exert antioxidant effects in glial cells [49], [51]. Conversely, Hsp90 has been implicated in neurodegenerative diseases, as it is found in protein aggregates and cellular inclusion bodies in Parkinson's disease (PD) [52]. In certain conditions, Hsp70 and Hsp90 act antagonistically, with Hsp70 affording protection and Hsp90 accelerating damage, such as in polyglutamine-mediated neurodegeneration and amyloid beta toxicity [52]. Congruent with these antagonistic roles, the Hsp90 inhibitor, geldanamycin, induces an overexpression of Hsp70 and protects against 1-methyl-4-phenyl-1,2,3,6-tetrahydropyridine (MPTP)-induced dopaminergic toxicity [53]. Due to these dual roles for Hsp90, and the shared pathways between neurodegenerative diseases and MeHg exposure, we explored whether MeHg activated or inhibited Hsp90. Hsp70 has been shown to be up-regulated in the cerebellum of mice exposed to MeHg, which may represent a protective response [54]. MeHg decreases glutathione peroxidase (GPx) and thioredoxin reductase (TrxR) activities through protein-thiol modification. These modifications often target the proteins for degradation, potentially mediated by Hsp70 chaperone activity. Increased Hsp70 in mouse cerebellum was correlated with decreased GPx1, GPx4, and TrxR1 protein levels [54]. Up-regulation of Hsp70 by MeHg is conserved in nonvertabrates; MeHg treatment increases HSP-4, the nematode homologue to Hsp70, in *Caenorhabditis elegans* [55], highlighting an important conserved response to the metal. We have observed that in primary cultured rat astrocytes that MeHg decreased Hsp90 levels in a concentration- and time-dependent manner (Figure 1). The down regulation of Hsp90 is unique to MeHg treatment as both chick embryos and rats treatment with HgCl_2_ show increased Hsp90 protein levels [56], [57]. These contrasting data suggest that Hsp90 responds differently to inorganic and organic Hg species. In addition to decreasing Hsp90 protein expression, we have shown that MeHg alters Hsp90 activity, as there are critical reactive cysteines found in the vicinity of Hsp90's ATP binding site that are potential targets for MeHg protein adduction and/or MeHg-induced oxidative stress.

PGES/p23 is an important Hsp90 client protein involved in MeHg toxicity. Previously, we have shown that MeHg increases the activity of cPLA2 in both neurons and astrocytes, causing an increase in AA [33], [58]. AA is converted into PGH_2_ by the actions of cyclooxygenases and peroxidase, which is a precursor for both isoprostanes and PGE_2_. Here we show that astrocytes release PGE_2_ in response to MeHg, in agreement with previous reports [47]. Depletion of GSH by BSO prevented PGE_2_ release, suggesting that MeHg-dependent release of PGE_2_ is redox-dependent. Astrocytes released significantly less PGE_2_ in response to MeHg when pretreated with the Hsp90 antagonist, GA (Figure 2). GA binds to the ATP binding site on Hsp90, inhibiting its ATP-dependent chaperone function, however GA does not prevent binding of Hsp90 to client proteins or disrupt existing Hsp90/client protein complexes [59]. Indeed, MeHg was shown to increase the interaction between Hsp90 and PGES/p23 in activated and resting astrocytes (Figure 3). Since Hsp90 chaperone function antagonism resulted in decreased PGE_2_ release following MeHg exposure, our data suggest that despite the loss of Hsp90 protein expression by MeHg, Hsp90 is capable of forming a complex with its client protein, PGES/p23. Excess PGE_2_ promotes Ca^2+^-dependent glutamate release from cultured astrocytes and brain slices, which combined with blocked glutamate reuptake by MeHg-induced AA release, results in markedly increased levels of extracellular glutamate and excitotoxicity [4], [60], [61].

Activation of the NMDA receptor increases intracellular concentration of Ca^2+^ and activates NOSs, which are also client proteins of Hsp90 [22], [62]. As astrocytes express nNOS and iNOS [63], [64], we investigated NOS activation as a second measure of the effect of MeHg on Hsp90 client protein binding. MeHg increased levels of both nNOS and iNOS in activated astrocytes, with a greater increase noted for nNOS (Figure 4). Additionally, MeHg increased the protein-protein interaction between nNOS and Hsp90 (Figure 4). The increase in nNOS and iNOS expression was associated with increased NO synthesis in response to MeHg treatment, suggesting that MeHg activates NOS. Additionally, we found that MeHg treatment decreased cGMP levels, the second messenger mediating downstream vasoactive NO signaling (Figure 5). Thus there appears to be an uncoupling of NO release from NO-dependent signaling in the presence of MeHg. Inhibition of the NO-cGMP signaling pathway has been observed in 3-month-old rats exposed to MeHg and polychlorinated biphenols (PCB126 and PCB153), leading to learning deficits (Piedrafita et al., 2008). NO has a number of important biochemical and physiological functions in the CNS, including neurotransmission, learning, regulation of glycolytic enzymes, pain perception, immune function and vascular regulation. Conversely, excessive NO release is cytotoxic. Over activation of glutamate receptors associated with cerebral ischemia and other excitotoxic processes results in massive release of NO [65]. NO mediates cellular toxicity by damaging critical metabolic enzymes and by reacting with •O_2_^−^ to form the even more potent peroxynitrite (ONOO^−^) [40]. High levels of NO are associated with inflammatory, neurodegenerative and cardiovascular/ischemic pathologies [66]. Several *in vitro* studies show that NO produced by iNOS in astrocytes mediates neuronal cell death after excitotoxic injury [67], [68], [69]. In our studies, both O_2_^−^ and H_2_O_2_ were significantly increased following MeHg exposure (Figure 6). Increases in NO and ONOO^−^ combined with Ca^2+^ overload damages the mitochondrial electron transport chain, resulting in reduced ATP formation, additional •O_2_^−^ formation, and cytochrome *c* release, all of which initiate a damaging cascade leading to neuronal death.

Taken together this study demonstrates for the first time that Hsp90 is an intermediary in MeHg toxicity. While MeHg decreases Hsp90 protein content from astrocytes following prolonged exposure, Hsp90 remains capable of interacting with its client proteins, PGES/p23 and nNOS, which generate increased amounts of PGE_2_, NO, and ROS in the presence of MeHg. Both of these pathways converge on glutamate signaling, mitochondrial dysfunction, and excitotoxic cell death.
